# Whole Genome Analyses Accurately Identify *Neisseria* spp. and Limit Taxonomic Ambiguity

**DOI:** 10.3390/ijms232113456

**Published:** 2022-11-03

**Authors:** May Khoder, Marwan Osman, Issmat I. Kassem, Rayane Rafei, Ahmad Shahin, Pierre Edouard Fournier, Jean-Marc Rolain, Monzer Hamze

**Affiliations:** 1 Laboratoire Microbiologie, Santé et Environnement (LMSE), Doctoral School of Sciences and Technology, Faculty of Public Health, Lebanese University, Tripoli 1300, Lebanon; 2 Institut de Recherche pour le Développement (IRD), Microbes, Evolution, Phylogénie et Infection (MEPHI), Faculté de Médecine et de Pharmacie, Aix Marseille Université, 13005 Marseille, France; 3 Cornell Atkinson Center for Sustainability, Cornell University, Ithaca, NY 14853, USA; 4 Department of Public and Ecosystem Health, College of Veterinary Medicine, Cornell University, Ithaca, NY 14853, USA; 5 Center for Food Safety, Department of Food Science and Technology, University of Georgia, Griffin, GA 30223-1797, USA

**Keywords:** *Neisseria* spp., identification, whole genome sequencing, taxonomy

## Abstract

Genome sequencing facilitates the study of bacterial taxonomy and allows the re-evaluation of the taxonomic relationships between species. Here, we aimed to analyze the draft genomes of four commensal *Neisseria* clinical isolates from the semen of infertile Lebanese men. To determine the phylogenetic relationships among these strains and other *Neisseria* spp. and to confirm their identity at the genomic level, we compared the genomes of these four isolates with the complete genome sequences of *Neisseria gonorrhoeae* and *Neisseria meningitidis* and the draft genomes of *Neisseria flavescens*, *Neisseria perflava*, *Neisseria mucosa*, and *Neisseria macacae* that are available in the NCBI Genbank database. Our findings revealed that the WGS analysis accurately identified and corroborated the matrix-assisted laser desorption ionization-time of flight (MALDI-TOF) species identities of the *Neisseria* isolates. The combination of three well-established genome-based taxonomic tools (in silico DNA-DNA Hybridization, Ortho Average Nucleotide identity, and pangenomic studies) proved to be relatively the best identification approach. Notably, we also discovered that some *Neisseria* strains that are deposited in databases contain many taxonomical errors. The latter is very important and must be addressed to prevent misdiagnosis and missing emerging etiologies. We also highlight the need for robust cut-offs to delineate the species using genomic tools.

## 1. Introduction

Historically, bacterial speciation has relied on a combination of phenotypic characteristics such as cultural characteristics and growth requirements, staining properties using Gram and Ziehl–Neelsen staining, morphology, motility, ultrastructure and chemical composition of the cell wall and outer membrane, metabolic pathways, and protein composition [1]. However, new parameters were adopted over time, particularly chemotaxonomy, genomic DNA-DNA hybridization (*is*DDH), GC% content, and numerical taxonomy [2]. Among the genotypic parameters, sequencing of the 16S rDNA gene has made a notable impact on bacterial taxonomy via the reclassification of many species or the identification of new species [3]. While 16S rDNA gene sequencing and *is*DDH were among the fundamental molecular taxonomic tools for many decades, they still suffered from many limitations. For example, 16S rDNA gene sequence similarity thresholds do not apply to multiple genera [4], the multiple rRNA operons in a single genome may exhibit nucleotide variations [5], and some of the 16S rDNA gene copies may be acquired by horizontal gene transfer, which may distort taxa relationships in phylogenetic trees [6]. Recently, advances in whole genome sequencing (WGS) have facilitated a better identification and classification of bacterial species, allowing the re-evaluation of taxonomic relationships between species [7,8,9]. Therefore, whole genome analysis provides a prime opportunity to identify and evaluate isolates belonging to *Neisseria*, a genus that encompasses notoriously hard-to-differentiate species.

The *Neisseria* genus contains 34 species (https://lpsn.dsmz.de/genus/neisseria (accessed on 2 November 2022)) that are Gram-negative diplococci, and many are harmless commensal inhabitants of the human and animal mucosal and dental surfaces. However, this genus also includes two significant human pathogens, *Neisseria gonorrhoeae* and *Neisseria meningitidis*, which can cause very different diseases, including gonorrhea and infrequently disseminated infections, and meningitis and septicemia, respectively [10]. Conventionally, *Neisseria* spp. are classified based on their phenotypic and biochemical properties. However, these techniques are not entirely effective in assigning isolates to species groups, which clearly would affect diagnosis and treatment. Therefore, genetic techniques were proposed for more accurate species identification and to explore the relationships between the *Neisseria* spp. [11].

Previously, we identified numerous isolates from the semen of infertile Lebanese men as *N. gonorrhoeae* using the biochemical assay, API^®^-NH (analytical profile index of *Neisseria* and *Haemophilus*, bioMérieux, Marcy l’Etoile, France). While confirming the identities of these isolates using advanced and other commonly used techniques, we discovered notable discrepancies between the identification approaches [8]. Consequently, we recognized this as an opportunity to evaluate *Neisseria* speciation discrepancies in our isolates using WGS. When comparing the sequences of *Neisseria* genomes that are deposited in databanks, we observed some misidentification errors in some of those present in the National Center for Biotechnology Information (NCBI) Genome database. Therefore, our current study aimed to analyze all complete genome sequences of *N. gonorrhoeae* and *N. meningitidis* and the draft genomes of *Neisseria flavescens*, *Neisseria perflava*, *Neisseria mucosa*, and *Neisseria macacae* that are available in the NCBI Genome database as well as the draft genomes of four Lebanese commensal *Neisseria* clinical isolates to confirm their identity and determine the phylogenetic relationships among these species at the genomic level. Subsequently, we aimed to shed light on the taxonomic problems prevalent in public databases and the pressing need for an update of the *Neisseria* genus.

## 2. Results

### 2.1. Species Identification

Using the API^®^-NH biochemical assay, all the strains isolated from the semen of the infertile Lebanese men (R19, R20, R21, and R23) were identified as *N. gonorrhoeae*. However, the matrix-assisted laser desorption ionization-time of flight (MALDI-TOF) analysis yielded completely different results, identifying R19, R21, and R23 as *N. flavescens* and R20 as *N. mucosa*. After performing 16S rDNA gene sequencing analysis, all the isolates were identified as *Neisseria* spp. but the identity of species could not be resolved.

### 2.2. Genome Sequencing and Genome Properties of the Lebanese Isolates

The draft genome of *N. flavescens* R19 consisted of 34 contigs (Accession number GCA_900654165) containing 2,207,472 bp and a GC content of 49.2%. According to the Prokka annotation, R19 harbored 2160 predicted genes, including 2091 protein-coding genes and 69 RNAs identified as 54 tRNA, 2 rRNA, 2 tmRNA, and 11 miscellaneous other RNA (misc_RNA) (Table 1). A total of 21 proteins were associated with virulence, including a type IV secretion system protein, iron-regulated ABC transporter ATP-binding protein, and major outer membrane protein PIB.

The draft genome of *N. mucosa* R20 consisted of 123 contigs (Accession number GCA_900654175) containing 2,541,217 bp with a GC content of 51%. Roughly, out of 2358 predicted genes, 2288 were protein-coding genes and 70 were RNAs including 54 tRNA, 2 rRNA, 1 tmRNA, and 13 misc_RNA. A total of 16 proteins were associated with virulence, including a type IV secretion system protein, trifunctional thioredoxin/methionine sulfoxide reductase, and catalase.

The draft genome of *N. flavescens* R21 harbored 2,268,952 bp and consisted of 36 contigs with a GC content of 49% (Accession number GCA_900654185). Additionally, R21 was predicted to harbor 2207 genes, including 2121 protein-coding genes and 86 RNAs as follows: 55 tRNA, 2 rRNA, 1 tmRNA, and 28 misc_RNA. A total of 21 proteins were associated with virulence, including a type IV secretion system protein, fatty acid efflux system protein FarB, and twitching motility protein PilT.

The draft genome of *N. flavescens* R23 consisted of 2,194,968 bp and 79 contigs with a GC content of 49.4% (Accession number GCA_900654195). Of 2206 predicted genes, 2100 were protein-coding genes and 106 were RNAs identified as 52 tRNA, 3 rRNA, 1 tmRNA, and 50 misc_RNA. A total of 21 proteins were found to be associated with virulence, including a type IV secretion system protein, fatty acid efflux system protein FarB, hemoglobin haptoglobin utilization protein HpuAB, and twitching motility protein PilT.

The major features of the *Neisseria* isolates’ genomes are summarized in Table S1, whereas their virulence factors are detailed in Table S2. Antibiotic resistance genes (ARGs) were not found in these four isolates.

### 2.3. Genome Comparison between the Lebanese Isolates and Other Neisseria Strains from the NCBI GenBank Database

The genomes of the four Lebanese isolates were compared to 128 available *Neisseria* genomes recovered from NCBI. Roughly, the NCBI *Neisseria* genomes had an average length of 2.16 Mb. The *N. perflava* strain UMB0210 (NZ_PKJP01000001.1) had the smallest genome with 2.13 Mb. and *N. perflava* strain CCH6-A12 (LSII01000021.1) had the largest one with 3.78 Mb. The GC content of genomes was an average of 51.61%, varying from 48.75% for *N. flavescens* strain CD-NF2 to 68.68% for *N. perflava* strain CCH6-A12.

For *N. flavescens* R19, the *is*DDH values ranged from 65.7% with N. flavescens CDNF3 to 30.9% with *N. meningitidis* MC58, 29.7% with *N. gonorrhoeae* FA1090, 28.9% with *N. mucosa* ATCC 19696, 24.3% with *N. perflava* CCH10H12, and 16.4% with *N. perflava* CCH6A12. Notably, relatively high *is*DDH values of 64.2% were also obtained with *N. perflava* UMB0023 as well as with *N. perflava* UMB0210 (Table 2).

As for *N. flavescens* R21, the *is*DDH values ranged from 65.1% with *N. flavescens* CDNF3 and 57.1% with *N. flavescens* NCTC8263 and *N. flavescens* NRL30031H210 to 0% with *N. perflava* CCH6A12. The lowest values were also obtained with other species such as *N. meningitidis* MC58 (31.2%), *N. gonorrhoeae* FA1090 (29.9%), *N. perflava* CCH10H12 (29.4%), and *N. mucosa* ATCC 19696 (29.3%). As noted previously, relatively high *is*DDH values were also found with *N. perflava* UMB0023 (62.6%) and *N. perflava* UMB0210 (62.6%).

Similarly, for *N. flavescens* R23, *is*DDH values were high with *N. flavescens* SK114 (69.9%), *N. flavescens* NCTC8263 (60.8%), and *N. flavescens* NRL30031H210 (60.8%). Relatively low values were noted with *N. meningitidis* MC58 (31.6%), *N. mucosa* ATCC 19696 (31.2%), *N. perflava* CCH10H12 (30.7%), *N. gonorrhoeae* FA1090 (30.1%), and *N. perflava* CCH6A12 (0%). Notably, relatively high values were observed with *N. perflava* UMB0210 (58.3%) and *N. perflava* UMB0023 (58.2%).

Regarding *N. mucosa* R20, relatively high *is*DDH values were found with *N. mucosa* C2004002444 (76.4%) and *N. mucosa* ATCC19696 (58.9%). However, low isDDH values of 28.8% and 29.2% were also found respectively with two *N. mucosa* strains (C102 and C6A). Similarly, other *Neisseria* spp. yielded low *is*DDH values with *N. mucosa* R20; for instance, *N. macacae* ATCC33926 (54.2%), *N. gonorrhoeae* FA1090 (32.8%), and *N. meningitidis* MC58 (34.2%) (Table 3).

**Figure 1:**
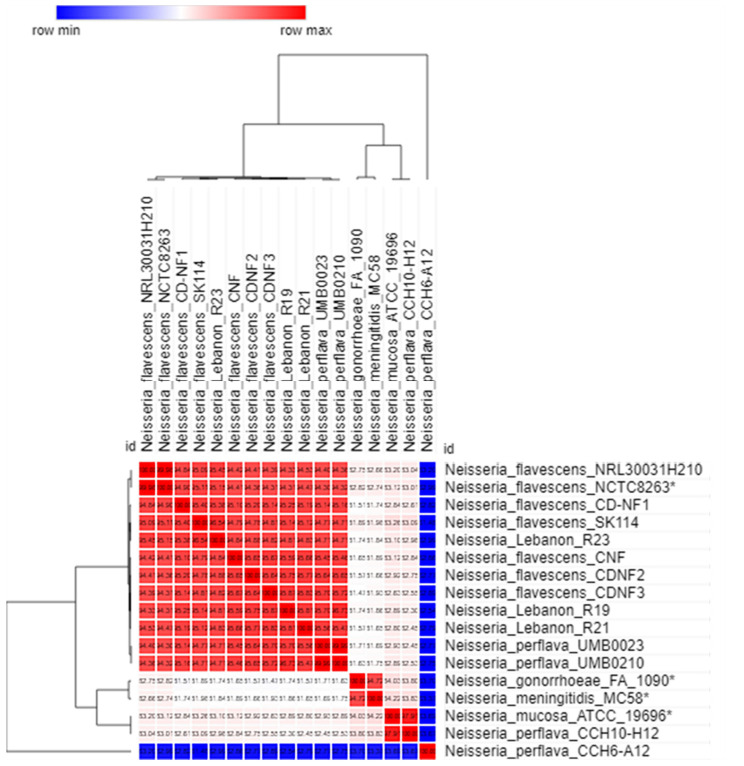
Functional heatmap of *Neisseria flavescens* and *Neisseria perflava* OrthoANI values. Reference genomes are marked by asterisk. Generation of the heatmap was done using Morpheus software (https://software.broadinstitute.org/morpheus/ (accessed on 2 November 2022)).

**Figure 2:**
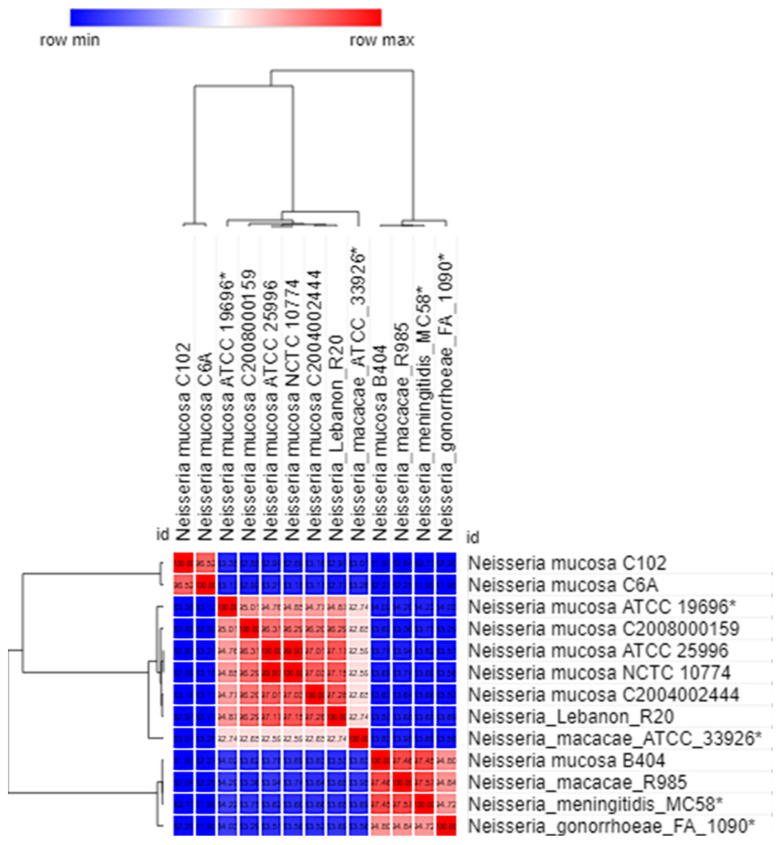
Functional heat-map of *Neisseria mucosa* and *Neisseria macacae* OrthoANI values. Reference genomes are marked by asterisk. Generation of the heatmap was done using Morpheus software (https://software.broadinstitute.org/morpheus/ (accessed on 2 November 2022)).

In order to verify the *is*DDH results, we complemented our previous analysis by estimating OrthoANI values represented by a heatmap (Figure 1 and Figure 2) between the Lebanese isolates and other *Neisseria* strains from the NCBI GenBank database. As a result, both *N. flavescens* R19 and *N. flavescens* R21 genomes exhibited their highest values (above 95–96%, the well-known cut-offs for species delimitation) with *N. flavescens* CDNF3 (of 95.85% and 95.83%, respectively). In contrast, *N. flavescens* R23 genome exhibited the highest value of 96.56% with *N. flavescens* SK114. *N. flavescens* R19, R21, and R23 showed the lowest OrthoANI values with *N. perflava* CCH6A12 (of 82.85%, 82.86%, and 83.6%, respectively). Additionally, *N. mucosa* R20 genome displayed high OrthoANI values of 97.18% with *N. mucosa* C2004002444 and 94.98% with *N. mucosa* ATCC19696, in contrast to the relatively lower values obtained with *N. macacae* ATCC33926 (92.97%), *N. mucosa* C6A (82.95%), *N. gonorrhoeae* FA1090 (84.39%%), and *N. meningitidis* MC58 (84.54%). Collectively, OrthoANI analysis corroborated the *is*DDH and MALDI-TOF identification of the Lebanese strains but potentially raised concerns about some taxonomic ambiguities in the genomes retrieved from the databases.

### 2.4. Pangenome and Phylogenetic Analysis of the Lebanese Isolates with Other Neisseria Strains Available in NCBI GenBank Database

**Figure 3:**
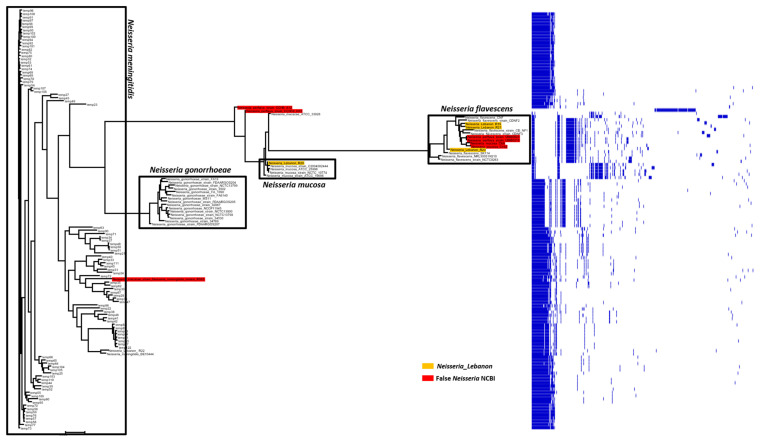
Pangenome tree of *Neisseria* isolated in Lebanon with 128 *Neisseria* spp. available in the NCBI GenBank database.

In order to confirm our previous results, pangenome analysis was performed. Roughly, the pangenome of the 128 NCBI *Neisseria* spp. contained 19,777 genes, including 88 conserved genes, 2218 shell genes shared by several species, and 17,314 cloud genes unique to one species. The phylogenetic tree resulting from the pangenome analysis confirmed the identities of our four Lebanese isolates, corroborating MALDI-TOF, *is*DDH, and OrthoANI analyses. Although some divergence between all the members of this genus was noted, the phylogenetic tree delineated four clusters encompassing *N. meningitidis*, *N. gonorrhoeae*, *N. mucosa*, or *N. macacae* isolates, one small cluster containing three species (*N. flavescens*, *N. perflava*, and *N. mucosa*), and one unclustered species (Figure 3). Interestingly, *N. perflava* UMB0023 and *N. perflava* UMB0210 were clustered together with *N. flavescens*, but *N. perflava* CCH6-A12 formed a phylogenetically distinct entity within *Neisseria*, while *N. perflava* CCH10-H12 clustered with *N. mucosa*. Furthermore, the pangenome analysis showed that the genomic sequences of *N. mucosa* C6A, *N. mucosa* C102, and *N. mucosa* B404 differed from other *N. mucosa* strains. Specifically, *N. mucosa* (C6A and C102) were not clustered with *N. mucosa* but with the *N. flavescens* group. Additionally, *N. mucosa* B404 and *N. macacae* R985 clustered together within the *N. meningitidis* group. Of note, the OrthoAni values of these two *N. mucosa* genomes surpassed the 95% cut-offs with *N. meningitidis* and were 97.45% (*N. mucosa* B404) and 97.57% (*N. macacae* R985), which potentially indicate that these strains were misidentified *N. meningitidis* species.

## 3. Discussion

*Neisseria* spp. are commonly misidentified in clinical laboratories because no adequate diagnostic tools are available for reliable identification of these species to date [12]. Although identification of these strains at the species level is generally not required at the clinical level, their misidentification distorts the results of epidemiological studies and has serious health and social consequences [13]. Commensal *Neisseria* spp. have been implicated in several cases of endocarditis, meningitis, sepsis, otitis, bronchopneumonia, and possibly genital tract diseases [14,15]. Therefore, when *Neisseria* spp. are isolated from clinical cases, microbiologists should be vigilant against dismissing them too readily as normal flora.

The first objective of our study was to unravel the identity of four Lebanese isolates recovered from semen samples and ambiguously identified as *N. gonorrhoeae* by the API^®^-NH biochemical assay. Corroborating the WGS analysis, MALDI-TOF gave the most accurate and comparable identification results in comparison to biochemical and 16S rDNA gene-based identification, highlighting its usefulness for the identification of commensal *Neisseria* spp. in routine diagnosis [12,13]. In another study, MALDI-TOF was found sufficient to be used as a single method for *Neisseria* identification with excellent performance in *N. gonorrhoeae* identification, but a careful interpretation was needed with *N. meningitidis* and commensal *Neisseria* spp. isolated from genital and oropharyngeal samples [16]. However, other studies suspected that the number of reference spectra in the MALDI-TOF database was insufficient, resulting in poor discriminatory power for closely related non-pathogenic *Neisseria* spp. [17,18]. To resolve this issue, some studies suggested to group *N. macacae* and *N. mucosa* isolates into the *N. mucosa* category and *N. flavescens* and *N. perflava* into one category with *N. subflava* [10,19]. Therefore, analysis of large collections of *Neisseria* isolates should be done to update the MALDI-TOF databases and to precisely determine the method’s relevance for the identification of species in this genus.

In Lebanon, only two MALDI-TOF devices have been available for less than a year throughout the country, and the vast majority of clinical laboratories use biochemical tests to identify bacteria. Furthermore, according to our previous report, *Neisseria* spp. are significantly present in the semen of infertile Lebanese men [12], suggesting a potential new role of these bacteria in the development of infertility in men in this region. Consequently, accurate diagnosis is essential to understand the epidemiology and etiology of the different *Neisseria* spp. to determine and treat *Neisseria* urogenital infections.

WGS represents today a valid tool for the taxonomic description and speciation of bacterial isolates [8,20]. *Neisseria* genus has benefited sparingly from the ongoing revolution of WGS, and nearly most of the genomic work focused on the two most clinically relevant *Neisseria* spp., *N. gonorrhoeae* and *N. meningitidis* [21,22,23], especially for outbreak detection [24] and disease and antimicrobial resistance surveillance [25,26,27]. The availability of WGS for *N. gonorrhoeae* rapidly increased due to the rise in multidrug-resistant gonococci which has provided a renewed impetus to resolve this global health threat [25]. Yet, the taxonomy of this genus remains a problem with a lot of ambiguity on species boundaries for non-*meningitidis* and non-*gonorrhoeae Neisseria* spp. [10]. In fact, species assignments for *N. meningitidis* and *N. gonorrhoeae* are currently well established [11], but many other species such as *N. perflava*, *N. macacae*, and *N. mucosa* require further attention. Additionally, recombination, which is considered high in the *Neisseria* genus, could have many distorting effects on *Neisseria* taxonomy where many mosaic genomes are regarded as “fuzzy species” or incipient species [28]. Despite the limited number of available genomic studies, they showed the extent of ambiguities in the current *Neisseria* classification scheme. For example, *Neisseria sicca* and *N. mucosa* are found to be very similar gnomically and can be considered variants of one species [10]. Furthermore, *Neisseria polysaccharea* were considered closely related to *N. meningitidis*, *N. gonorrhoeae*, and *Neisseria lactamica* isolates, but they did not represent a monophyletic group [10]. Moreover, genome sequence analyses showed that *Neisseria oralis* is the same species as *N. mucosa* var. heidelbergensis [29]. Therefore, WGS studies are needed to facilitate resolving the identification and taxonomic conundrums of *Neisseria* spp.

For the first time, we report here the importance of genomic approaches to shed light on the taxonomic problems occurring in public databases and the need to revisit the taxonomy of *Neisseria* spp. Previous studies mainly used genomic data to infer ribosomal MLST-based *Neisseria* taxonomy [10,30]. In comparison, our study adopted three well-established genome-based taxonomic tools (*is*DDH, OrthoANI, and pangenomic analyses) to verify the identification and limit taxonomical errors. It was proposed that the 70% threshold for *is*DDH analysis (adopted for the wet lab DDH) is not a universal cutoff and does not apply to many genera [1]. Concerning the *Neisseria* genus, genomes of different species can share close *is*DDH values, which potentially confirms their genetic similarity and the difficulty of defining a universal cut-off (as the case for *N. perflava* and *N. flavescens*). To resolve this issue, we complemented our analysis by (1) calculating the Average Nucleotide Identity (ANI), which was considered a valid alternative to *is*DDH (with ANI values of 95–96% as cut-offs); and (2) constructing pangenome-based phylogenetic relationships. Indeed, the latter was found very useful to stratify two distinct *Klebsiella* subspecies (*K. pneumoniae* subsp. *ozaenae* and *K. pneumoniae* subsp. *rhinoscleromatis*) at the species level [31].

In this study, we analyzed four draft genomes of *N. perflava*. Our results indicated that the two *N. perflava* strains (UMB0023 and UMB0210) are genetically closely related to *N. flavescens* due to high OrthoANI (95.66% and 95.71%), *is*DDH values (64.2%), and a close clustering in the pangenome-based phylogenetic tree. Thus, these strains can be misidentified as *N. flavescens*. Notably, data from historical studies indicate that *N. perflava* is more closely related to *N. flavescens* than other *Neisseria* spp. and could be incorporated into the species *N. subflava* [10,19]. For this, additional genomic work must be done in the near future to unravel the real taxonomic position of *N. perflava* species; either as *N. flavescens* closely related species or *N. flavescens* synonymous species. Furthermore, we found that *N. perflava* CCH6-A12 is an unclustered species that probably does not belong to the *Neisseria* genus, because it has no core genome in common with other *Neisseria* spp. and shows very low OrthoANI values (65.31% with *N. meningitidis* M25070 and 65% with *N. mucosa* ATCC 19696). Moreover, *N. perflava* CCH10-H12 did not cluster with *N. perflava* but with *N. mucosa* group sharing high *is*DDH (80.7%) and OrthoANI values (97.7%).

Among the six analyzed *N. mucosa* genomes, two genomes (C6A and C102) did not cluster with *N. mucosa* but with the *N. flavescens* group, sharing relatively high OrthoANI values (95.1% and 95%). In addition, *N. mucosa* B404 and *N. macacae* R985 clustered together within the *N. meningitidis* group (see the results section for more detail). This highlights the extent of ambiguity in *Neisseria* taxonomy and how identification and or taxonomy errors can prevail and propagate even in databases.

## 4. Materials and Methods

### 4.1. Isolation of Strains

Four strains of *Neisseria* (R19, R20, R21 and R23) were isolated on polyViteX chocolate agar (PVX, bioMérieux, Marcy l’Etoile, France) from semen samples of infertile Lebanese men at Nini Hospital in Tripoli, Lebanon. The colonies were first identified as *N. gonorrhoeae* by API^®^-NH (bioMérieux, Marcy l’Etoile, France). After that, MALDI-TOF Biotyper (Bruker Daltonics, Bremen, Germany) was used to confirm species identification. The spectra of these isolates were imported into the MALDI-TOF Bruker Biotyper software system (version 2.0) and analyzed by standard pattern matching (default parameter settings). Additionally, 16S rDNA gene sequencing analysis was performed on these isolates [32].

### 4.2. Genomic DNA Preparation and Genome Sequencing

The DNA was isolated and purified using the EZ1 DNA Tissue Kit (BioRobot EZ1 Advanced XL instrument, Qiagen, Hilden, Germany) following the manufacturer’s instructions. Genomic DNA of the four Lebanese isolates was sequenced using the MiSeq Technology (Illumina Inc, San Diego, CA, USA). Briefly, the genomic DNA was quantified by the Qubit assay with the high sensitivity kit (Life technologies, Carlsbad, CA, USA) and 0.2 µg/µL of the DNA was used for sequencing. The DNA was fragmented and amplified by a limited PCR (12 cycles), introducing dual-index barcodes and sequencing adapters. After purification on AMPure XP beads (Beckman Coulter Inc, Fullerton, CA, USA), the libraries were normalized and pooled for sequencing on the Illumina MiSeq platform (Illumina Inc., San Diego, USA). Paired-end sequencing and automated cluster generation with dual indexed 2 × 250-bp reads were performed for 40 h run. Total information of 8.2 Gb was obtained from a 1,207,000/mm^2^ cluster density with a cluster passing quality control filters of 89.3% (10507.2 passed filtered reads). The mate pair library was prepared with 1.5 µg of genomic DNA using the Nextera Mate-Pair Illumina guide. The genomic DNA sample was simultaneously fragmented and tagged with a mate-pair junction adapter.

### 4.3. Genome Annotation and Genome Comparisons

The draft genomes were assembled by the A5 pipeline [33], organized by mauve alignment and annotated by Prokka [34] and RAST [35], as described previously. The virulence factors were determined by ABRICATE (https://github.com/tseemann/abricate/ (accessed on 2 November 2022)). Furthermore, the ARGs were identified through BLAST search in the Bio-Edit interface against the ARGannot database [36] under moderately stringent conditions (e-value of 10^−5^). The putative ARGs were further verified through a web BLAST search using the NCBI non-redundant nucleotide database. In parallel, we retrieved from NCBI the genome sequences of 128 strains of *Neisseria*, including *N. gonorrhoeae* (15 complete genomes), *N. meningitidis* (91 complete genomes), *N. flavescens* (7 draft genomes), *N. perflava* (4 draft genomes), *N. mucosa* (1 complete genome and 8 draft genomes), and *N. macacae* (2 draft genomes) (Table S1). In addition, to estimate the similarity between the genome of the Lebanese *Neisseria* isolates and the other genomes, the Genome-to-Genome Distance Calculator (GGDC, http://ggdc.dsmz.de (accessed on 2 November 2022)) with formula 2 was used, because it calculates the in silico *is*DDH values. The mean levels of relatedness between the genome sequences were measured using OrthoAni (Orthologous Average Nucleotide Identity) (https://www.ezbiocloud.net/tools/orthoani (accessed on 2 November 2022)). A pairwise comparison between the genome of *Neisseria* spp. was generated using OrthoAni values in Morpheus software (https://software.broadinstitute.org/morpheus/ (accessed on 2 November 2022)), which displayed them graphically as heatmaps. Moreover, the pangenomes of the Lebanese isolates together with the 128 NCBI *Neisseria* pangenomes were analyzed using the Roary pangenome pipeline on the Galaxy web-based platform (https://usegalaxy.org.au./ (accessed on 2 November 2022)). A reference genome was used for each species in this analysis (*N. gonorrhoeae* FA1090, *N. meningitidis* MC58, *N. mucosa* ATCC 19696, *N. flavescens* NCTC8263, and *N. macacae* ATCC33926).

## 5. Conclusions

*Neisseria* isolates need to be accurately identified, because some strains may be misidentified as pathogenic species, while other strains can occasionally be isolated from unusual sites and must be correctly identified and verified to establish clinical relevance and emerging strains. We compared the core/pan-genome of different *Neisseria* genomes and found that the genus *Neisseria* contains many taxonomical errors in the genome databases and requires a reexamination to remove ambiguity and misidentifications. Additionally, there is a need for robust cut-offs (e.g., *is*DDH values) to facilitate further the use and benefit of genomic analysis. While WGS represents a good solution for the identification of *Neisseria* spp., it should be noted that financial barriers remain a major limitation against the use of these technologies, particularly in developing countries. However, based on our data, *Neisseria* infections require an in-depth examination in these countries because of the high probability of the emergence of new disease-causing isolates.

## Figures and Tables

**Table 1:** Genomic annotation of the four Lebanese *Neisseria* isolates.

Genome	Isolate	Species	Isolation Date	Genes (N)	CDS (N)	RNA (N)
R19	CMUL013	*N. flavescens*	2014	2160	2091	tmRNA: 2 rRNA: 2 tRNA: 54 misc_RNA: 11
R20	CMUL032	*N. mucosa*	2015	2358	2288	tmRNA: 1 tRNA: 54 rRNA: 2 misc_RNA: 13
R21	CMUL057	*N. flavescens*	2016	2207	2121	tmRNA: 1 tRNA: 55 rRNA: 2 misc_RNA: 28
R23	CMUL078	*N. flavescens*	2017	2206	2100	tmRNA: 1 tRNA: 52 rRNA: 3 misc_RNA: 50

CMUL, Lebanese University bacterial bank; CDS, coding potential or protein coding sequence.

**Table 2:** Comparison of *N. flavescens* R19, R21, and R23 with related *Neisseria* spp. using GGDC, formula 2 (DDH estimates based on identities/high scoring segment pair (HSP) length).

	Genome	1	2	3	4	5	6	7	8	9	10	11	12	13	14	15
**1**	*Neisseria_flavescens_NRL30031H210*	100														
**2**	*Neisseria_flavescens_SK114*	60.6	100													
**3**	*Neisseria_flavescens_CD-NF1*	59.4	62.9	100												
**4**	*Neisseria_flavssescens_CDNF2*	57.4	59.6	61.4	100											
**5**	*Neisseria_flavescens_CDNF3*	57.2	59.8	60.8	64.9	100										
**6**	*Neisseria_ flavescens_CNF*	57	59.3	60.9	63.7	63.3	100									
**7**	*Neisseria_flavescens_NCTC8263* *	93.1	60.6	59.4	57.4	57.1	57.1	100								
**8**	*Neisseria_gonorrhoeae_FA_1090* *	31.9	30.3	29.7	29.5	29.4	29.8	32	100							
**9**	*Neisseria_meningitidis_MC58* *	33.6	31.7	31	30.5	31.4	31.5	33.8	57.6	100						
**10**	*Neisseria_mucosa_ATCC_19696* *	30.2	31.5	29.1	29.9	29.2	29.6	30.6	33.5	35	100					
**11**	*Neisseria_perflava_CCH6-A12*	14.5	14.7	14.8	14.7	0	14.5	14.5	15.6	0	13.6	100				
**12**	*Neisseria_perflava_CCH10-H12*	29.8	30.7	29.5	28.9	29.1	29.2	30.2	33.3	34.8	80.7	14.9	100			
**13**	*Neisseria_perflava_UMB0023*	56.4	58.8	60.6	63.6	64.2	62.2	56.5	30	30.6	29.3	14.5	29.1	100		
**14**	*Neisseria_perflava_UMB0210*	56.5	58.9	60.6	63.6	64.2	62.2	56.5	30	30.6	29.3	14.5	29	99.2	100	
**15**	*Neisseria_Lebanon_*R19	56.6	59.1	61.6	64.9	65.7	63.6	56.6	29.7	30.9	28.9	16.4	29.3	64.2	64.2	100
**15**	*Neisseria_Lebanon_*R21	57.1	59.1	60.5	64.4	65.1	63.3	57.1	29.9	31.2	29.3	0	29.4	62.6	62.6	100
**15**	*Neisseria_Lebanon_*R23	60.8	69.9	62.6	59.8	59.5	58.7	60.8	30.1	31.6	31.2	0	30.7	58.2	58.3	100

Accession number of: *N. flavescens* R19 is GCA_900654165, *N. flavescens* R21 is GCA_900654185, and *N. flavescens* R23 is GCA_900654195. Reference genomes are marked by asterisk.

**Table 3:** Comparison of *N. mucosa* R20 with related *Neisseria* spp. using GGDC, formula 2 (DDH estimates based on identities/HSP length).

	Genome	1	2	3	4	5	6	7	8	9	10	11	12	13	14
**1**	*Neisseria_mucosa*_C102	100													
**2**	*Neisseria_mucosa*_ATCC_19696 *	29.5	100												
**3**	*Neisseria_mucosa*_ATCC_25996	29	58.4	100											
**4**	*Neisseria_mucosa*_C6A	69.4	30.2	29.4	100										
**5**	*Neisseria_mucosa*_C2004002444	29	58.7	75.3	29.3	100									
**6**	*Neisseria_mucosa*_C2008000159	44.4	59.5	67.8	29.2	67.6	100								
**7**	*Neisseria_mucosa*_B404	28.9	34.6	34	30.7	33.9	33.7	100							
**8**	*Neisseria_mucosa*_NCTC_10774	29.2	58.4	89.4	29.6	74.4	67.5	34.2	100						
**9**	*Neisseria_mucosa*_CCH7-A10	28.5	63.4	0	27.1	100	100	0	59.7	100					
**10**	*Neisseria_macacae*_ATCC_33926 *	29.4	63.5	53.7	29.7	54	54.5	34.3	53.9	52.6	100				
**11**	*Neisseria_macacae*_R985	30.5	34.6	33.9	30.6	34.1	33.4	76.6	34	0	33.9	100			
**12**	*Neisseria_meningitidis*_MC58 *	30.9	35	34.3	31.1	34.6	34.1	76.3	34.5	35.8	34.2	78	100		
**13**	*Neisseria_gonorrhoeae*_FA_1090 *	30	33.5	32.9	30	32.9	33.2	58.2	33.1	33.9	33.4	58.4	57.6	100	
**14**	*Neisseria_Lebanon*_R20	28.8	58.9	74.3	29.2	76.4	68	33.6	74.3	60.5	54.2	33.6	34.2	32.8	100

Accession number of *Neisseria mucosa* R20 is GCA_900654175. Reference genomes are marked by asterisk.

## Data Availability

Not applicable.

## Supplementary Materials

The supporting information can be downloaded at: https://www.mdpi.com/article/10.3390/ijms232113456/s1.
